# P-1087. Predictors of Mortality and Outcomes in Patients with Infections Due to Vancomycin-Resistant Enterococcus faecium

**DOI:** 10.1093/ofid/ofaf695.1282

**Published:** 2026-01-11

**Authors:** José C Rodríguez-Jiménez, Jaina Y Aldana-Vázquez, Alfredo Ponce-de-León, María F González-Lara, Bernardo A Martínez-Guerra

**Affiliations:** Instituto Nacional de Ciencias Médicas y Nutrición Salvador Zubirán, Mexico City, Distrito Federal, Mexico; Universidad Marista de Mérida, Mexico City, Distrito Federal, Mexico; Instituto Nacional de Ciencias Médicas y Nutrición Salvador Zubirán, Mexico City, Distrito Federal, Mexico; Instituto Nacional de Ciencias Médicas y Nutrición Salvador Zubirán, Mexico City, Distrito Federal, Mexico; Instituto Nacional de Ciencias Médicas y Nutrición Salvador Zubirán, Mexico City, Distrito Federal, Mexico

## Abstract

**Background:**

Infections due to vancomycin-resistant *Enterococcus faecium* (VRE) represent major therapeutic challenges and carry a high mortality. Limited rapid diagnostic platforms and therapeutic options exist. Early identification of mortality predictors could guide appropriate timely treatment. We aimed to identify the factors associated with 90-day all-cause mortality in patients with VRE infections.

**Methods:**

We conducted a retrospective cohort study that included all admitted patients with VRE infections from January 1, 2013, to December 31, 2023. Patients were follow-up for 90 days. The primary outcome was 90-day all-cause mortality after VRE diagnosis. Secondary outcomes included length of stay, mechanical ventilation, ICU admission, renal replacement therapy (RRT), and infection relapse during follow-up. Bivariate and multivariate Cox-proportional hazards models were constructed to identify factors independently associated with 90-day all-cause mortality.

**Results:**

A total of 184 patients were included. Death occurred in 65 (35%) cases. The most frequent infection site was intraabdominal (84, 45%). Frequent comorbidities were immunosuppression (106, 58%), arterial hypertension (45, 25%), hematologic malignancy (40, 22%) and diabetes mellitus (39, 21%). Appropriate therapy was administered in 145 (79%) cases. The median length of stay in survivors was 42 days (IQR 28-65). Mechanical ventilation, ICU admission, and RRT during follow-up occurred in 8/144 (6%), 12/143 (8%) and 7/162 (4%), respectively. Infection relapse occurred in 19/184 (10%) cases.

In the multivariate analysis, prior RRT (aHR 2.33, 95%CI 1.06-5.14), hematologic malignancy (aHR 3.46, 95%CI 1.60-7.50), and septic shock at diagnosis (aHR 5.37, 95%CI 2.99-9.66) were associated with all-cause death. Antibiogram-guided appropriate therapy was associated with a lower risk of death (aHR 0.30, 95%CI 0.16-0.55).

**Conclusion:**

Renal replacement therapy, hematologic malignancy, and septic shock are associated with worse prognosis. Antibiogram-guided appropriate therapy is associated with a lower risk of death. Recognition of the prognostic factors could assist in improving patient care. Therapeutic options must be widely available and promptly prescribed when necessary.

**Disclosures:**

All Authors: No reported disclosures

